# TDP‐43 pathology and functional deficits in wild‐type and ALS/FTD mutant cyclin F mouse models

**DOI:** 10.1111/nan.12902

**Published:** 2023-04-10

**Authors:** Annika van Hummel, Miheer Sabale, Magdalena Przybyla, Julia van der Hoven, Gabriella Chan, Astrid F. Feiten, Roger S. Chung, Lars M. Ittner, Yazi D. Ke

**Affiliations:** ^1^ Dementia Research Centre, Macquarie Medical School, Faculty of Medicine, Health and Human Sciences Macquarie University Sydney New South Wales Australia; ^2^ Biomedical Center (BMC), Division of Metabolic Biochemistry, Faculty of Medicine Ludwig‐Maximilians‐Universität München Munich 81377 Germany; ^3^ Centre for Motor Neuron Disease Research, Macquarie Medical School, Faculty of Medicine, Health and Human Sciences Macquarie University Sydney New South Wales Australia

**Keywords:** amyotrophic lateral sclerosis, cyclin F, frontotemporal dementia, mouse models, TDP‐43, ubiquitination

## Abstract

**Aims:**

Amyotrophic lateral sclerosis (ALS) is characterised by a progressive loss of upper and lower motor neurons leading to muscle weakness and eventually death. Frontotemporal dementia (FTD) presents clinically with significant behavioural decline. Approximately 10% of cases have a known family history, and disease‐linked mutations in multiple genes have been identified in FTD and ALS. More recently, ALS and FTD‐linked variants have been identified in the *CCNF* gene, which accounts for an estimated 0.6% to over 3% of familial ALS cases.

**Methods:**

In this study, we developed the first mouse models expressing either wild‐type (WT) human CCNF or its mutant pathogenic variant S621G to recapitulate key clinical and neuropathological features of ALS and FTD linked to CCNF disease variants. We expressed human CCNF WT or CCNF^S621G^ throughout the murine brain by intracranial delivery of adeno‐associated virus (AAV) to achieve widespread delivery via somatic brain transgenesis.

**Results:**

These mice developed behavioural abnormalities, similar to the clinical symptoms of FTD patients, as early as 3 months of age, including hyperactivity and disinhibition, which progressively deteriorated to include memory deficits by 8 months of age. Brains of mutant CCNF_S621G mice displayed an accumulation of ubiquitinated proteins with elevated levels of phosphorylated TDP‐43 present in both CCNF_WT and mutant CCNF_S621G mice. We also investigated the effects of CCNF expression on interaction targets of CCNF and found elevated levels of insoluble splicing factor proline and glutamine‐rich (SFPQ). Furthermore, cytoplasmic TDP‐43 inclusions were found in both CCNF_WT and mutant CCNF_S621G mice, recapitulating the key hallmark of FTD/ALS pathology.

**Conclusions:**

In summary, CCNF expression in mice reproduces clinical presentations of ALS, including functional deficits and TDP‐43 neuropathology with altered CCNF‐mediated pathways contributing to the pathology observed.

## Graphical abstract

The first CCNF mouse models were developed in the present study using adeno‐associated viruses to express wild‐type (WT) human CCNF or its mutant pathogenic variant S621G with GFP as control. These CCNF mice reproduce clinical presentations of ALS, including functional deficits and TDP‐43 neuropathology with altered CCNF‐mediated pathways contributing to the pathology observed.

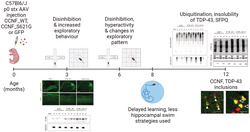

**Key points**

- CCNF WT and CCNF^S621G^ mice display behavioural and functional deficits reminiscent of frontotemporal dementia (FTD) and amyotrophic lateral sclerosis (ALS) including hyperactivity, disinhibition and progressive memory deterioration.
- CCNF WT and CCNF^S621G^ mice recapitulate classical neuropathological features of the disease including the presence of ubiquitinated proteins such as the splicing factor proline and glutamine‐rich (SFPQ) protein, valosin‐containing protein (VCP) and elevated levels of phosphorylated TDP‐43.
- Key proteins implicated in FTD and ALS such as CCNF, VCP, SFPQ and TDP‐43 all mechanistically converge on common molecular pathways in relation to FTD/ALS pathogenesis.

## INTRODUCTION

Amyotrophic lateral sclerosis (ALS) and frontotemporal dementia (FTD) have overlapping clinical and pathological presentations in which the molecular and pathogenic basis remains poorly understood. ALS is characterised by a progressive loss of upper and lower motor neurons leading to characteristic muscle weakness, wasting and eventually death. Over 50% of ALS patients develop cognitive impairment, and behavioural variant FTD is seen in approximately 15% of all ALS patients. FTD, on the other hand, presents clinically with either significant behavioural deterioration or predominant language decline and atrophy of the frontal and/or temporal cortex as the predominant histopathological feature [1]. Ubiquitinated protein aggregates containing the TAR DNA‐binding protein 43 (TDP‐43) are the key characteristic pathological feature of both ALS and FTD patients. Up to 15% of ALS patients are also diagnosed with FTD, and segregation of both ALS and FTD may be seen within families. Approximately 10% of ALS patients have a known family history of the disease, and variants in several genes such as *SOD1*, *FUS*, *TARDBP*, *UBQLN2*, *PFN1*, *OPTN*, *VCP*, *MATR3*, *TUBA4A* and *C9ORF72* have been discovered and account for the majority of familial ALS and 5% of sporadic ALS cases [2, 3, 4, 5, 6, 7, 8, 9, 10, 11]. More recently, ALS and FTD‐linked variants have been identified in the *CCNF* gene, which accounts for an estimated 0.6 to over 3% of familial ALS cases [12, 13, 14].

*CCNF* encodes the 786 amino acid protein Cyclin F (CCNF). CCNF is a member of the F‐box protein family, characterised by the presence of an F‐box motif, which acts as the substrate recognition component of the Skp1‐Cul1‐F‐box (SCF) E3 ubiquitin ligase complex responsible for mediating ubiquitination of target proteins. CCNF plays an important role in maintaining homeostasis through the timely degradation of damaged and unwanted proteins [15].

Cellular models utilising CCNF expression had been extensively used to assess key mechanisms involved in understanding the pathobiology of disease‐linked CCNF variants. Expression of S621G mutant CCNF (CCNF^S621G^) in cells resulted in excessive CCNF‐mediated ubiquitination [16]. Proteomic analysis identified the presence of disrupted caspase 3‐mediated cell death and viability pathways, suggesting a toxic gain‐of‐function associated with CCNF^S621G^ expression [17]. Expression of CCNF^S621G^ in Neuro‐2A cells resulted in a specific increase in levels of Lys48‐ubiquitylated proteins, but not Lys63‐ubiquitylated proteins [18]. Proteomic analysis identified the clustering of these Lys48‐ubiquitylated proteins to the autophagy pathway [18].

Zebrafish were used to study disease‐linked CCNF functions for the first time in vivo [17]. CCNF^S621G^ expression led to increased cleaved caspase‐3‐mediated cell death as well as abnormal axonal outgrowth with concomitantly reduced motor function in zebrafish [17]. However, no accumulation of ubiquitinated proteins was observed in this model, which was attributed to the brief period of mutant CCNF^S621G^ expression.

In this study, we developed the first mouse models expressing either wild‐type (WT) human CCNF or its mutant pathogenic variant S621G, which recapitulate key clinical presentations of FTD and neuropathological features of both ALS and FTD linked to CCNF disease variants. We expressed human CCNF WT or CCNF^S621G^ throughout the murine central nervous system by intracranial delivery of adeno‐associated virus (AAV) to achieve widespread brain delivery via somatic brain transgenesis. These mice developed behavioural abnormalities similar to the clinical symptoms of FTD patients. Brains of CCNF mice displayed accumulation of ubiquitinated proteins, elevated levels of phosphorylated TDP‐43 and cytoplasmic TDP‐43 inclusions, recapitulating the key hallmark of FTD/ALS pathology. Behavioural and neuropathological changes were more pronounced in CCNF_S621G than CCNF_WT mice.

## MATERIALS AND METHODS

### AAV plasmid and production

N‐terminally V5‐tagged human WT and mutant CCNF with the point mutation S621G were separately cloned into the multiple cloning site of an AAV vector (pAM‐CAG) under the CAG‐promoter for neuronal expression [19]. All plasmids were amplified and propagated in VB UltraStable competent cells (VectorBuilder).

Packaging of AAV vectors was performed as previously described using the AAV.PHP.B capsid [20, 21]. Briefly, 293 T cells were seeded in complete DMEM (Sigma) with 10% FBS, and the medium was changed to IMDM (Sigma) with 5% FBS 3 h prior to transfection. Cells were transfected with an AAV vector carrying the transgene (pAAV‐CAG‐V5‐CCNF WT, pAAV‐CAG‐V5‐CCNF S621G or control pAAV‐CAG‐EGFP), pFdelta6 as helper plasmid and AAV‐PHP.B plasmid containing rep and cap sequences using polyethyleneimine‐Max (PEI‐Max, Polysciences). Cells and supernatant were harvested 72 h post‐transfection and clarified in 40% PEG8000/2.5 M NaCl to a final concentration of 8% PEG8000/0.5 M NaCl at 4°C for 2 h and centrifuged at 2,000*g* for 30 min. The combined precipitate was subsequently treated with sodium deoxycholate (0.5% final concentration) and benzonase (~500 U) at 37°C for 40 min. After the addition of NaCl, incubation at 56°C for 40 min and freeze–thaw, the solution was centrifuged for 30 min at 5,000*g* at 4°C. Supernatants were purified using iodixanol gradient by ultracentrifugation (475,900*g* for 2 h at 18°C). AAV particles were concentrated in PBS in an Amicon 100 kDa 15 ml concentrator at 5,000*g* at 4°C. Titres were determined by quantitative polymerase chain reaction (qPCR). AAV titres were (in viral genomes per ml): pAAV‐CAG‐V5‐CCNF WT (2.16 × 10 [14]), pAAV‐CAG‐V5‐CCNF S621G (1.59 × 10 [14]) and pAAV‐CAG‐EGFP (2.17 × 10 [14]).

### Mice and AAV injections

Time‐mated C57Bl/6 mice were obtained from ARC Perth and allowed to give birth. AAV injections were carried out on pups at postnatal day 0 to 1 (P0‐P1) whereby 1.2 μl/site (6 × 10^10^ viral particles) of AAV particles was injected at five sites bilaterally into the brains (total 30 × 10^10^ viral particles) of cryo‐anaesthetized neonatal mice as described [20].

All mice were housed on a 12‐h light/dark cycle with access to standard chow and water ad libitum. Both male and female mice were used throughout this study. All experiments were approved by the Macquarie University animal care and ethics committee.

### Histological analysis

Mice were anaesthetized and transcardially perfused at either 3, 8 or 12 months of age with PBS. Brains were removed and the hemispheres separated. One hemisphere of the brain was immersion‐fixed in 4% paraformaldehyde for immunohistochemical analysis as previously described [22, 23], and the other hemisphere was sub‐dissected and snap‐frozen in liquid nitrogen for biochemical analysis. Fixed brains were processed using an automated system (Excelsior, Thermo, USA), embedded in paraffin and sagittally sectioned at the level of the mid‐hippocampus into 3‐μm‐thick sections using a microtome (Thermo, USA). All staining was done in Sequenza staining racks for standardisation using previously reported protocols [24]. The following primary antibodies were used: pan‐TDP‐43 (10782‐2‐AP, Proteintech), POU3F2 (ab94977, Abcam), GFAP (G9269, Sigma), EGFP (ab184601, Abcam), Cyclin F (sc‐515207, Santa Cruz) and VCP (sc‐20799, Santa Cruz). Microscopy was performed with an Olympus BX51 (United States) epi‐fluorescence microscope equipped with an XM10 MONO camera, or sections were scanned with an Axio Scan Z1 automated slide scanner (Zeiss).

### Behavioural, memory and motor testing

C57Bl/6 mice injected with WT CCNF (*n* = 19), CCNF^S621G^ (*n* = 14) or EGFP (*n* = 11) AAV underwent motor (rotarod and hanging wire test) and behavioural testing (elevated plus maze and open field) at 3 and 6 months of age followed by cognitive testing (Morris Water Maze) at 8 months of age.

### Rotarod

The motor performance of mice was determined using a Rota‐Rod (Ugo Basile) in acceleration mode (5–60 rpm) over 120 s. The longest time each mouse remained on the turning wheel out of five attempts per session was recorded.

### Elevated plus maze

The Elevated Plus maze (Ugo Basile) consisted of two open and two closed arms (each 35 cm × 5.5 cm), as well as a central platform (5.5 cm × 5.5 cm), elevated 60 cm above the ground. Mice were acclimatised to the room for 1 h prior to testing and then placed on the centre platform facing an open arm and recorded for 5 min. Videos were analysed using the AnyMaze software (Stoelting Co., IL, USA).

### Open field

Mice were placed at the periphery of a 40 cm × 40 cm Perspex box in an enclosed cupboard and videoed for 10 min. Videos were analysed using the AnyMaze software, and the box was divided into an outer and inner zone (the inner zone was a 17.5 cm × 17.5 cm^2^ in the centre of the box).

### Hanging wire test

Mice were placed on a wire mesh and allowed to hang upside down for a maximum of 3 min; latency to fall off was recorded (longest time out of two attempts).

### Morris water maze

The apparatus consisted of a 1.5‐m‐diameter tank with a 40‐cm‐high Perspex platform (diameter 10 cm), which was placed roughly 20 cm from the edge of the wall. The tank was filled to 0.5–1 cm above the surface of the platform, and a non‐toxic, acrylic‐based paint was added to the water to obscure the platform. Four signposts with different shapes were placed equidistant around the pool as visual cues. Mice were acclimatised to the room for 1 h prior to testing each day. Days 1–6 consisted of an acquisition phase, in which mice were placed in the quadrant opposite the platform at one of four starting positions and given 60 s to locate the hidden platform. Mice that failed to find the hidden platform were guided to the escape platform, and all mice remained on the platform for an additional 60 s before being removed from the maze. Mice had four trials per day, each starting from a different position, and the order of starting positions was altered each day. On the seventh day, the platform was removed, and the mice were given 30 s to explore the pool (probe trial). On the eighth day, the platform was placed back in the pool with a flag attached, and visual cues were removed from outside of the pool, to ensure that all mice had normal vision. Videos were analysed using the AnyMaze software. Accordingly, trace plots obtained for each swim were classified visually based on previously published paradigm [25]. Briefly, swim patterns were scored as follows: 1, thigmotaxis; 2, random swim; 3, scanning; 4, chaining; 5, directed search; 6, focal search; and 7, direct swim. Based on this scoring scheme, 1–3 reflect non‐spatial hippocampal‐dependent, while search strategies 4–7 are categorised to reflect spatial hippocampal learning.

### RIPA/UREA solubility extraction

Sequential extraction to determine the solubility of TDP‐43 has been previously described [26]. Briefly, 50–100 mg of brain tissue was homogenised and sonicated in a modified RIPA buffer (50 mM tris [pH 8], 150 mM NaCl, 0.1% Na‐dodecyl sulphate, 0.5% Na‐deoxycholate, 1% Nonidet™ P 40 substitute, 5mM EDTA and protease inhibitor cocktail [cOmplete™, Roche]). Lysates were incubated for 30 min, rotating at 4°C before they were subjected to a 1 h, 50,000*g* centrifugation step. The supernatant was collected as the RIPA‐soluble fraction. Two further homogenisation/centrifugation washes were carried out to remove any remnants of RIPA‐soluble proteins before the RIPA‐insoluble pellet was further extracted and sonicated in Urea buffer (7 M urea, 2 M thiourea, 4% CHAPS and 30 mM tris [pH 8.5]). Protein extracts were analysed by immunoblotting as previously described [27]. The following antibodies were used: mouse anti‐ubiquitin (FK2) (catalogue #BML‐PW8810, Enzo Life Sciences), pan‐TDP‐43 (10782‐2‐AP, Proteintech), GFAP (G9269, Merck), CCNF (sc‐515207, Santa Cruz Biotechnology), human TDP‐43 (60019‐2‐Ig, Proteintech), phospho‐TDP‐43 S403/S404 (TIP‐PTD‐P05, Cosmo Bio), phospho‐TDP‐43 S409/410 (CAC‐TIP‐PTD‐P02, Cosmo Bio), phospho‐TDP‐43 S409 (CAC‐TIP‐PTD‐P03, Cosmo Bio), total ubiquitin (Z0458, Agilent‐Dako), ubiquitin FK2 (ST1200, Merck), GAPDH (MAB374, Merck), SFPQ (ab38148, abcam) and S100β (ab52642, abcam).

### Statistical analysis

All statistical analyses were performed using Graphpad Prism 9 software using ANOVA (Tukey's multiple comparison test) or binomial distribution (for MWM swim path analysis only). *P* values below 0.05 were considered significant. All values are presented as the mean and standard error of the mean.

## RESULTS

### Generation of WT CCNF and mutant CCNF (S621G) mouse models by AAV‐mediated gene expression

**FIGURE 1:**
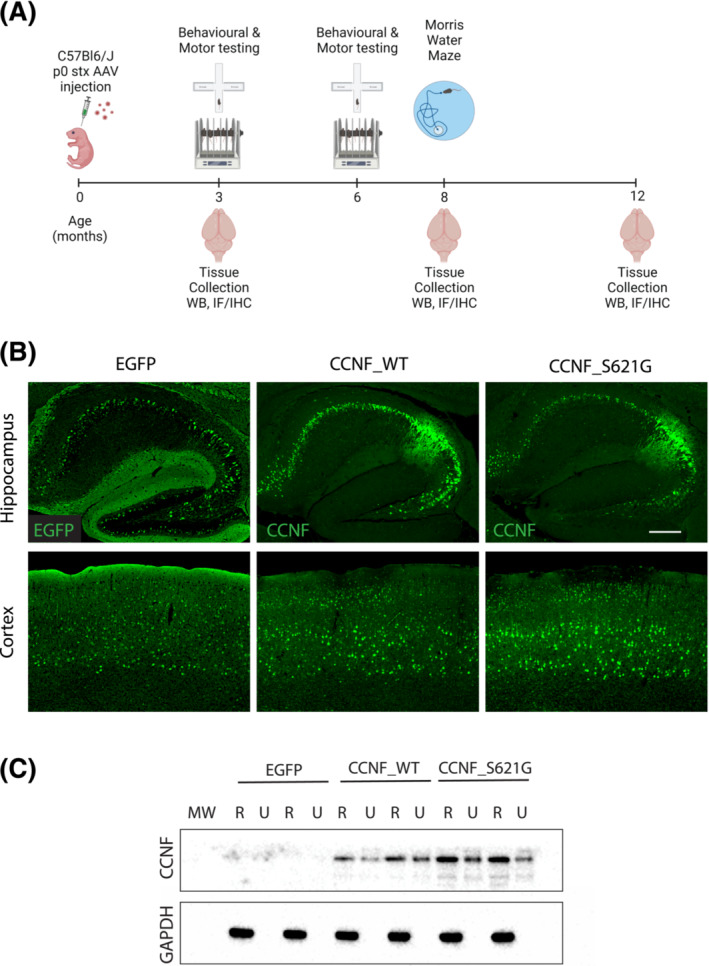
Widespread CCNF expression in the hippocampus and cortex. (A) Schematic of the experimental protocol. Wild‐type mice were intracranially injected at birth with control EGFP, CCNF_WT or CCNF_S621G AAV to achieve neuronal expression in the brain. Mice underwent behavioural testing at 3, 6 and 8 months of age with tissue collected for histological and biochemical analysis at 3, 8 and 12 months of age. (B) Immunofluorescence with an anti‐human CCNF antibody, which only recognises the AAV‐expressed CCNF, revealed widespread expression in the hippocampus and cortex of CCNF_WT and CCNF_S621G mice. Scale bar 250 μm. (C) Expression levels were further confirmed via immunoblotting of RIPA (R)/UREA (U) extracts of 3‐month‐old control EGFP, CCNF_WT and CCNF_S621G cortices.

Adeno‐associated viral vectors to convey neuronal expression of either EGFP (=control), non‐mutant WT CCNF (CCNF_WT) or CCNF harbouring the pathogenic S621G mutation (CCNF_S621G) were intracranially injected in neonatal C57Bl/6 mice. These mice were functionally analysed using a series of behavioural and motor tests at 3, 6 and 8 months of age. Tissue was subsequently collected for biochemical and histological analysis (Figure 1A). Expression of EGFP, CCNF_WT and CCNF_S621G were widespread in the cortex and hippocampus (Figure 1B). Levels of transgenic CCNF expression in mouse brains at 3 months of age were comparable between CCNF_WT and mutant CCNF_S621G mice (Figure 1C). Both WT and mutant CCNF were present in the urea‐soluble fraction, indicative of insoluble CCNF accumulation.

### Expression of WT and mutant CCNF S621G causes behavioural and cognitive deficits

**FIGURE 2:**
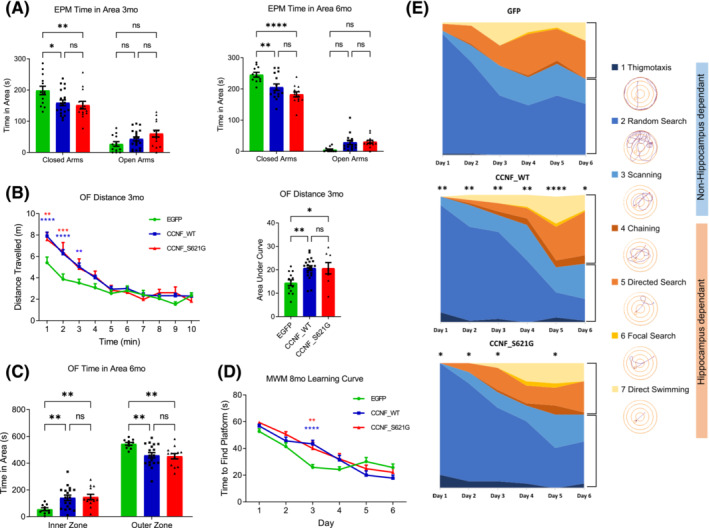
Cognitive and behavioural deficits in CCNF_WT and CCNF_S621G mice. (A) Time spent in closed and open arms of the elevated plus maze (EPM) at 3 months of age (3 months; left graph; *p < 0.05, **p < 0.01; ns, non‐significant; two‐way ANOVA [Tukey's multiple comparisons] *F* [2, 22] = 4.406) and 6 months of age (right graph; **p < 0.01, ****p < 0.0001; two‐way ANOVA [Tukey's post hoc] *F* [2, 36] = 10.33) for control EGFP (green), CCNF_WT (blue) and CCNF_S621G (red). (B) Distance travelled per minute in 10‐min open field (OF) test at 3 months (left graph; **p < 0.01, ***p = 0.0001, ****p < 0.0001; two‐way ANOVA [Tukey post hoc] *F* [18, 342] = 3.730, red * = S621G vs. EGFP, blue * = WT vs EGFP) and area under the curve for the first 5 min of the OF at 3 months (right graph; *p < 0.05, ^**^ *p* < 0.01, one‐way ANOVA [Tukey post hoc] *F* [2, 38] = 6.864). (C) Time spent in the inner and outer zones of the OF at 6 months (**p < 0.01; two‐way ANOVA [Tukey's post hoc] *F* [2, 38] = 5.798). (D) Memory testing at 8 months of age in the Morris water maze (MWM) with mean latency from 4 swims to find an escape platform on individual days of the acquisition phase over 6 days (**p < 0.01, ****p < 0.0001; two‐way ANOVA [Tukey post hoc] *F* [10, 250] = 4.613, red * = S621G vs. EGFP, blue * = WT vs. EGFP). (E) Swim path strategies used by mice during the acquisition phase of the MWM for control EGFP, CCNF_WT and CCNF_S621G mice (*p < 0.05, **p < 0.01, ****p < 0.0001). Categories 1–3 are considered non‐hippocampus‐dependent, and categories 4–7 are considered hippocampus‐dependent; example traces for each category are shown.

CCNF_WT, CCNF_S621G and control EGFP mice underwent a battery of motor and behavioural tests at 3, 6 and 8 months of age (Figure 1A). The elevated plus maze (EPM) paradigm was used to determine if CCNF_WT and CCNF_S621G mice exhibit changes in anxiety‐related behaviour. At 3 and 6 months of age, WT and mutant CCNF‐injected mice spent significantly less time in the closed arms compared to EGFP controls with a trend towards increased time spent in the open arms (Figure 2A), suggesting reduced anxiety or disinhibition. The total distances travelled during EPM testing were indistinguishable between the test cohorts (Figure S1A).

When exposed to a novel environment in the open field arena, 3‐month‐old CCNF_WT and CCNF_S621G mice displayed increased distance travelled in the first 5 min of the test (Figure 2B), suggesting increased activity, but no changes to the exploratory pattern (Figure S1B). Furthermore, while control EGFP preferentially explored the outer zone of the open field arena, 6‐month‐old CCNF_WT and CCNF_S621G mice spent significantly more time in the inner zone, further suggesting reduced anxiety (Figure 2C). Despite the altered exploration patterns in 6‐month‐old CCNF_WT and CCNF_S621G mice, their total distance travelled during testing was comparable (Figure S1C).

To determine if memory formation is compromised in CCNF_WT and CCNF_S621G mice, spatial memory was assessed using the Morris water maze (MWM) paradigm. Eight‐month‐old CCNF_WT and CCNF_S621G mice showed delayed learning during the memory acquisition phase, taking significantly longer to find the hidden platform at day 3 than EGFP controls (Figure 2D). At the same time, there were no differences in time spent in the platform quadrant or distance travelled during the probe trials between test cohorts (Figure S1D). A detailed analysis of the learning phase by categorising swim strategies into the hippocampus‐ or non‐hippocampus‐dependent patterns showed that CCNF_WT mice used significantly fewer hippocampus‐dependent swim strategies on days 1–6 and CCNF_S621G mice on days 1, 2, 3 and 5 compared to EGFP controls (Figure 2E).

Finally, motor performance was assessed in EGFP, CCNF_WT and CCNF_S621G mice by subjecting them to the accelerating rotarod and hanging wire tests. No differences between groups were observed in either rotarod (Figure S1E) or hanging wire tests (Figure S1F) at 3 or 6 months of age. Similarly, no differences in weight were observed in males or females between the groups at either 3 or 6 months of age (Figure S1G).

Taken together, CCNF_WT and CCNF_S621G mice presented with hyperactivity, reduced anxiety and impaired spatial memory acquisition, reminiscent of clinical symptoms of FTD [1] in the absence of any overt motor impairments.

### Loss of POU3F2 immunoreactivity in the cortex of aged mutant CCNF_S621G mice

**FIGURE 3:**
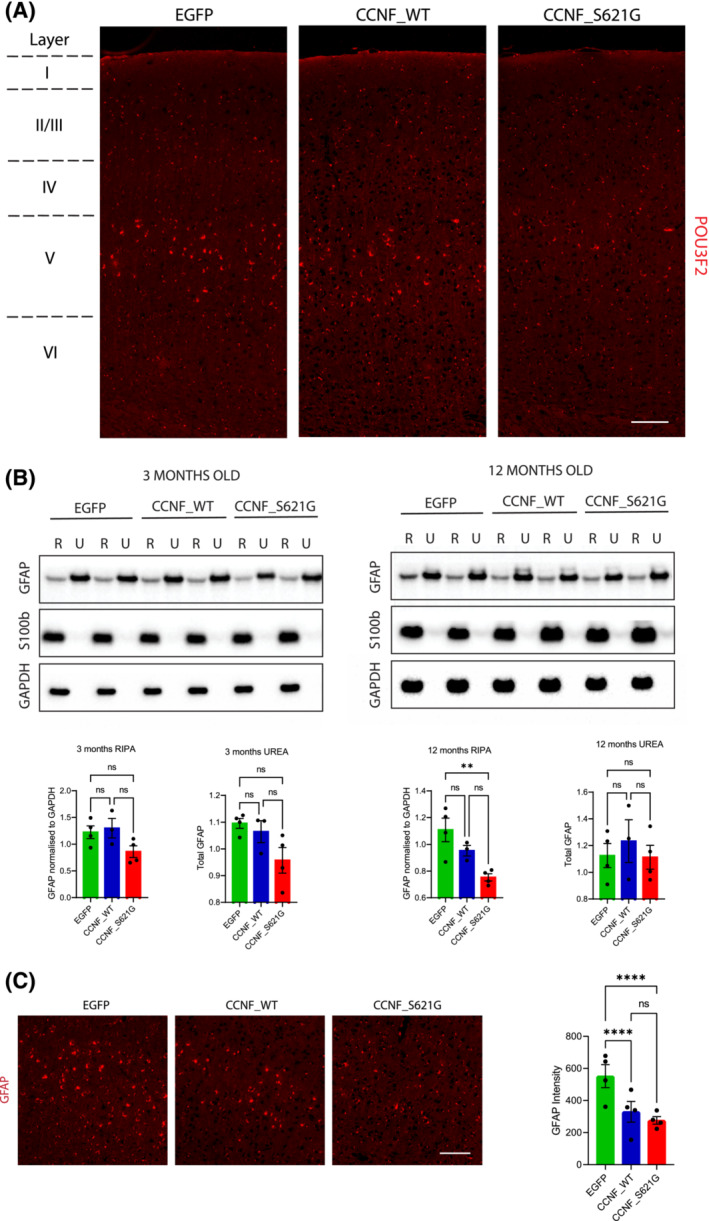
Loss of POU3F2 in cortical layer V occurs in the absence of gliosis. (A) Immunofluorescence with POU3F2 antibody with the corresponding cortical layers marked on the left. Scale bar 100 μm. (B) Immunoblotting of RIPA (R)/UREA (U) extracts with antibodies against GFAP for astrocyte activation, S100b for astrocyte levels and GAPDH as a housekeeping protein. Quantification of the respective bands (**p < 0.01; one‐way ANOVA [Tukey post hoc] *F* = 9.092). (C) Immunofluorescence with GFAP (red) antibody in control EGFP, CCNF_WT and CCNF_S621G cortex at 12 months of age with the corresponding quantification of overall fluorescence intensity provided on the right (****p < 0.0001; two‐way ANOVA [Tukey post hoc] F [6, 24] = 16.78). Scale bar 100 μm.

Next, we performed a neuropathological analysis of the brains of 3‐ and 12‐month‐old EGFP, CCNF_WT and CCNF_S621G mice. Macroscopically and histologically, there were no signs of overt degeneration with a normal appearance of the hippocampus (Figure 1B) and indistinguishable thickness of the cortex (Figure 3A) in CCNF_WT and CCNF_S621G mice compared to EGFP controls. However, further analysis of cortical neuronal layers using the marker for layer II/III/V, POU3F2, showed a reduction in its staining in cortical layer V of 12‐month‐old CCNF_S621G mice. POU3F2 staining in layers II and III was indistinguishable between EGFP, CCNF_WT and CCNF_S621G mice and between EGFP and CCNF_WT mice in layer V (Figure 3A). Astrogliosis is a common feature of neurodegenerative conditions and is frequently observed in mouse models of ALS [26, 28, 29]. Therefore, astroglial states in EGFP, CCNF_WT and CCNF_S621G mice were next assessed by the presence of GFAP‐positive cells in the cortex. Surprisingly, there was a significant reduction of GFAP levels in cortical RIPA‐fractions of 12‐month‐old CCNF_S621G mice compared to EGFP controls. The observed reduction was progressive, as GFAP levels were comparable at 3 months of age across the three lines, with only a trend towards reduction in CCNF_S621G mice (Figure 3B). Immunofluorescent staining of 12‐month‐old CCNF_S621G cortex further confirmed the reduction in GFAP immunoreactivity compared to EGFP controls (Figure 3C). This reduction in astroglial activation occurred independently of any change to the overall levels of the astroglial marker S100beta, which were comparable in EGFP, CCNF_WT and CCNF_S621G mice at both 3 and 12 months of age (Figure 3B).

In summary, we found a progressive reduction in astroglial activation in 12‐month‐old CCNF_S621G mice, concomitant with a reduction in POU3F2‐immunoreactivity in cortical layer V of these mice.

### Ubiquitination and insolubility of CCNF's interaction targets in CCNF mice

**FIGURE 4:**
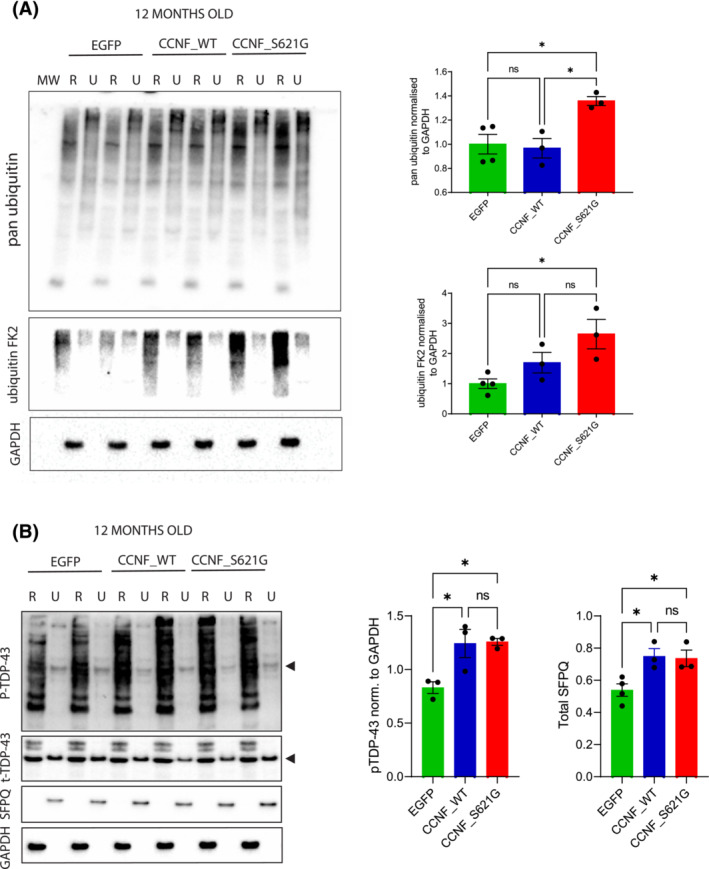
Ubiquitination, phosphorylation and insolubility in CCNF mice. (A) Immunoblotting of RIPA (R)/UREA (U) extracts of 12‐month‐old EGFP, CCNF_WT and CCNF_S621G with two different pan‐ubiquitination antibodies (pan‐ubiquitin, FK2) with GAPDH as a housekeeping protein. Quantification of pan‐ubiquitin and FK2 bands in the RIPA fraction normalised to GAPDH shown on the right (*p < 0.05; one‐way ANOVA [Tukey post hoc] *F* = 8.118). (B) Immunoblotting of RIPA (R) /UREA (U) extracts of 12‐month‐old EGFP, CCNF_WT and CCNF_S621G with antibodies against total TDP‐43 (t‐TDP‐43), phosphorylated TDP‐43 (pTDP‐43) at serine 409/410 and splicing factor proline and glutamine‐rich (SFPQ) with GAPDH as a housekeeping protein. Quantification of pTDP‐43 (normalised to GAPDH) in RIPA fraction and SFPQ bands in UREA fraction shown on the right with arrowheads depicting the TDP‐43 band (*p < 0.05; one‐way ANOVA [Tukey post hoc] *F* = 8.305 for pTDP‐43, *F* = 7.258 for SFPQ).

Aberrant protein phosphorylation and ubiquitination are hallmark features of ALS [30]. Using sequential protein extraction with buffers of increasing stringency (=RIPA followed by UREA buffer), we found elevated levels of ubiquitinated proteins only in cortices of CCNF_S621G mice but not CCNF_WT mice compared to EGFP controls (Figure 4A). In addition, we probed the effects of WT and mutant CCNF expression on their known interaction partners, TDP‐43 and the splicing factor proline and glutamine‐rich (SFPQ). Phosphorylation of TDP‐43 at serine 409/410 is a pathological feature present in all forms of TDP‐43 proteinopathies [31]. Consistent with this, we found an increase in the levels of TDP‐43 phosphorylated at serines 409/410 in both CCNF_WT and CCNF_S621G mice (Figure 4B). Furthermore, we analysed levels of SFPQ in these mice and found increased SFPQ in the UREA fraction of 12‐month‐old CCNF_WT and CCNF_S621G mice (Figure 4B).

In essence, elevated levels of TDP‐43 phosphorylation at pathological sites and accumulation of insoluble SFPQ were observed in both CCNF_WT and CCNF_S621G mice, while aberrant protein ubiquitination was only observed in CCNF_S621G mice.

### Cytoplasmic inclusions in CCNF_WT and CCNF_S621G mice

Cytoplasmic localisation of CCNF has previously been reported when overexpressed in cells [32]. Accordingly, some cytoplasmic localisation of CCNF was observed in the cortices of 3‐month‐old CCNF_WT mice in addition to its nuclear presence. This cytoplasmic CCNF was more pronounced in CCNF_S621G mice (Figure S2).

**FIGURE 5:**
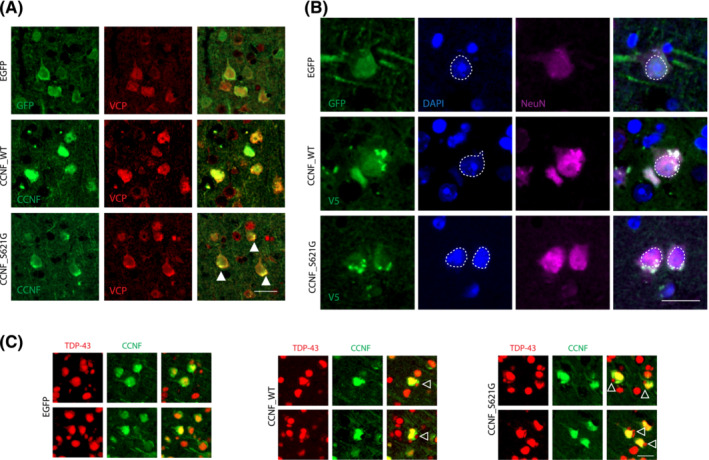
Cytoplasmic inclusions in CCNF mice. (A) Immunofluorescence with either GFP or CCNF coupled with double labelling with VCP antibodies in the cortices of 3‐month‐old control EGFP, CCNF_WT and CCNF_S621G. Closed arrows depict cytoplasmic inclusions containing both CCNF and VCP. Scale bar 25 μm. (B) Immunofluorescence with NeuN, DAPI and either GFP or V5 antibodies in the cortices of 12‐month‐old control EGFP, CCNF‐WT and CCNF_S621G. Dashed lines depict nuclear boundaries using DAPI. Scale bar 20 μm. (C) Immunofluorescence with TDP‐43 and CCNF antibodies in 12‐month‐old EGFP, CCNF_WT and CCNF_S621G cortices revealed cytoplasmic inclusions (open arrows) in CCNF_WT and CCNF_S621G mice. Scale bar 25 μm.

Mutations in the valosin‐containing protein (VCP) have previously been reported in ALS [33], and CCNF has been shown to directly interact with VCP, enhancing its activity [34]. Accordingly, we found CCNF colocalised with VCP in the nucleus and cytoplasm of neurons in CCNF_WT mice (Figure 5A). Interestingly, we observed the presence of cytoplasmic inclusions of CCNF that colocalised with VCP in CCNF_S621G mice (Figures 5A and S2). Co‐immunofluorescence labelling of NeuN, DAPI with either GFP or V5 confirmed the cytoplasmic localization of these CCNF inclusions in CCNF_WT and CCNF_S621G cortices (Figure 5B). This led us to next investigate whether expression of CCNF in mice leads to the altered subcellular localisation of TDP‐43. In line with ALS‐like pathology, we found cytoplasmic inclusions of TDP‐43 in the cortices of 12‐month‐old CCNF_WT and CCNF_S621G mice (Figure 5C). Interestingly, the co‐labelling of sections showed that the TDP‐43 inclusions did not colocalise with CCNF (Figure 5C).

Taken together, cytoplasmic inclusions, containing both CCNF and VCP, were observed in 3‐month‐old CCNF_WT and CCNF_S621G, which eventually led to cytoplasmic TDP‐43 aggregates at 12 months of age.

## DISCUSSION

To the best of our knowledge, our study is the first to generate mouse models of ALS with transgenic expression of CCNF in either its non‐mutant WT form or carrying the pathogenic ALS variant S621G using AAVs. CCNF_WT and CCNF_S621G mice developed progressive functional deficits with disinhibition and impaired spatial memory acquisition. While increased activity in the open field was observed as well in these mice, they lacked overt motor impairments. Loss of POU3F2 immunoreactivity in cortical layer V was present only in CCNF_S621G mice, and surprisingly, there was also a reduction in astroglial straining at 12 months of age. Increased protein ubiquitination was observed in 12‐month‐old CCNF_S621G mice with a concomitant increase in phosphorylated‐TDP‐43 and insoluble SFPQ. Finally, cytoplasmic inclusions containing CCNF and VCP were observed at 3 months, preceding TDP‐43 aggregates at 12 months in both CCNF_WT and CCNF_S621G mice. Why TDP‐43 pathology was not associated with increased astroglial responses as seen in mouse lines expressing mutant TDP‐43 [26, 35, 36, 37], but rather a decrease in cortical GFAP staining, remains to be understood. One possibility is that the TDP‐43 pathology resulting from increased CCNF expression in 12 months old mice reflects an early stage of neuropathology, and only advanced neuronal TDP‐43 deposition causes concomitant gliosis.

Several interaction partners of CCNF have been identified. These include proteins involved in cell cycle functions such as ribonucleoside‐diphosphate reductase subunit M2 (RRM2), nucleolar and spindle‐associated protein 1 (NuSAP1), centriolar coiled‐coil protein of 110 kDa (CP110), cell division control protein 6 homologue, stem‐loop binding protein, amongst others. In addition, CCNF has also been found to interact with other proteins such as TDP‐43, sequestosome‐1, valosin‐containing protein [34] and more recently, splicing factor proline and glutamine‐rich (SFPQ) [32]—all previously linked to ALS/FTD. Notably, overexpression of mutant CCNF S621G in HEK293 cells led to both aggregation and increased cytoplasmic localisation of SFPQ [32]. In line with this, we found an increase in the presence of insoluble SFPQ in 12‐month‐old CCNF_WT and CCNF_S621G mice. These findings recapitulate what is observed in human neuropathology with SFPQ identified in aggregates in cases of ALS [38] as well as in detergent‐insoluble fractions in FTLD cases [39].

CCNF expression has been previously shown to result in increased VCP ATPase activity, which in turn promotes cytoplasmic aggregation of TDP‐43 [34]. Here, we showed at 3 months of age that CCNF_S621G mice developed cytoplasmic inclusions containing both CCNF and VCP with some sparse inclusions present in CCNF_WT mice. We speculate that CCNF expression in mice (both WT and mutant S621G) resulted in increased VCP ATPase activity as previously reported [34], which in turn led to the cytoplasmic aggregation of TDP‐43 in both CCNF_WT and CCNF_S621G mice at 12 months of age. Alternatively, the aberrant ubiquitination induced by increased CCNF activity (due to transgenic overexpression) may have resulted in progressive insolubility of TDP‐43, and eventually in the formation of inclusions, similar to ubiquitinated TDP‐43 inclusions found in ALS, as TDP‐43 is a ubiquitylation substrate of the CCNF complex [40]. While CCNF would be required to confer ubiquitination, no direct interaction with CCNF is subsequently required to form TDP‐43 aggregates, and hence, no colocalization in inclusions was observed in the mice.

## CONCLUSIONS

In summary, non‐mutant and mutant CCNF expression in CNS neurons of mice is sufficient to reproduce aspects of the clinical presentation of ALS, including functional deficits and TDP‐43 neuropathology, making it a good candidate for developing and testing tailored therapies for ALS. Our data suggest that increased CCNF activity dysregulates specific pathways in vivo governed by its interaction partners and ubiquitination targets, including TDP‐43.

## AUTHOR CONTRIBUTIONS

YDK, AVH and LMI planned and designed the project. RC contributed to project discussions. AVH, AFF, MP and JVDH carried out behavioural and motor testing of mice. AVH analysed behavioural and motor testing data. MS carried out biochemical extractions and western blotting. MS and YDK analysed western blotting data. YDK and LMI made the figures and wrote the manuscript. All authors read and approved the final manuscript.

## CONFLICT OF INTEREST STATEMENT

The authors declare that they have no competing interests.

## ETHICS STATEMENT

All experiments were approved by the Macquarie University Animal Care and Ethics Committee.

## Supporting information


**Figure S1.** Behavioural and motor tests of 3‐and 6‐month‐old CCNF mice. (A) No difference between groups in distance travelled in the Elevated Plus maze (EPM) at 3 months of age (3mo) and 6mo. (B) No difference in time spent in the inner and outer zones in the open field (OF) test at 3mo. (C) No difference in distance travelled in the OF at 6mo. (D) No difference was observed between groups for time spent or distance travelled in the platform quadrant in the Morris water maze (MWM) probe trial. (E) No difference between groups on the accelerating rotarod test at 3mo and 6mo. (F) No difference between groups in the hanging wire test at 3mo and 6mo. (G) Comparable weights of males and females at 3mo and 6mo between the groups.

**Figure S2.** Cytoplasmic inclusions in CCNF mice. Immunofluorescence of either GFP or CCNF double labelled with VCP antibodies in the cortices of 3‐month‐old control EGFP, CCNF_WT and CCNF_S621G. Boxed areas are depicted at higher magnification in Figure 5A. Scale bar 50 μm.

## Data Availability

The data that support the findings of this study are available from the corresponding author upon reasonable request.
