# Supplementary figures and images for: TDP‐43 pathology and functional deficits in wild‐type and ALS/FTD mutant cyclin F mouse models
Source: Neuropathol Appl Neurobiol. 2023 Apr 10;49(2):e12902. doi: 10.1111/nan.12902 (PMC10946706; doi:10.1111/nan.12902)

Suppl Figure 1

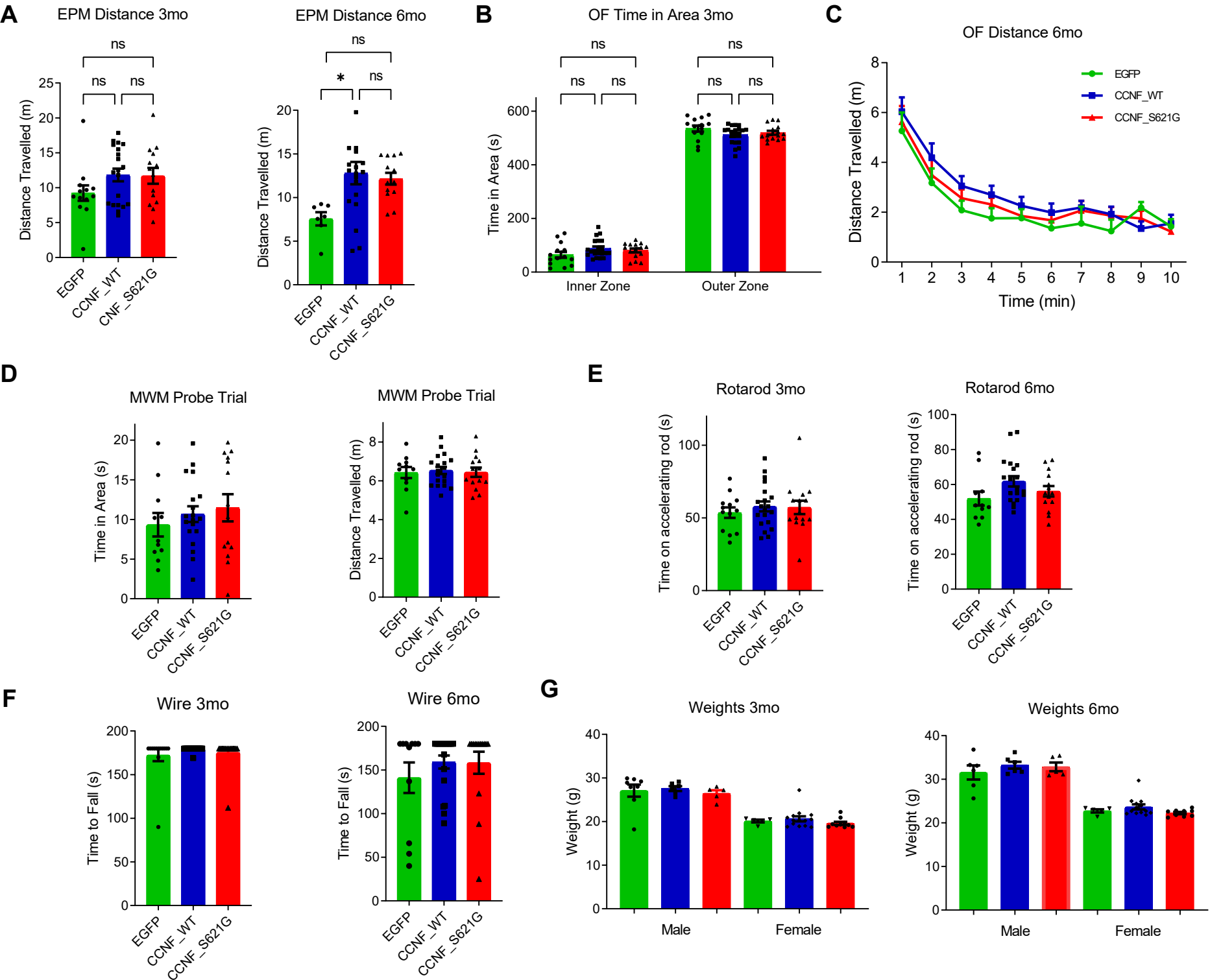

Supplement: Supplementary file 1 — Figure S1. Behavioural and motor tests of 3‐and 6‐month‐old CCNF mice. (A) No difference between groups in distance travelled in the Elevated Plus maze (EPM) at 3 months of age (3mo) and 6mo. (B) No difference in time spent in the inner and outer zones in the open field (OF) test at 3mo. (C) No difference in distance travelled in the OF at 6mo. (D) No difference was observed between groups for time spent or distance travelled in the platform quadrant in the Morris water maze (MWM) probe trial. (E) No difference between groups on the accelerating rotarod test at 3mo and 6mo. (F) No difference between groups in the hanging wire test at 3mo and 6mo. (G) Comparable weights of males and females at 3mo and 6mo between the groups. [file NAN-49-0-s002.pdf]

Suppl Figure 2

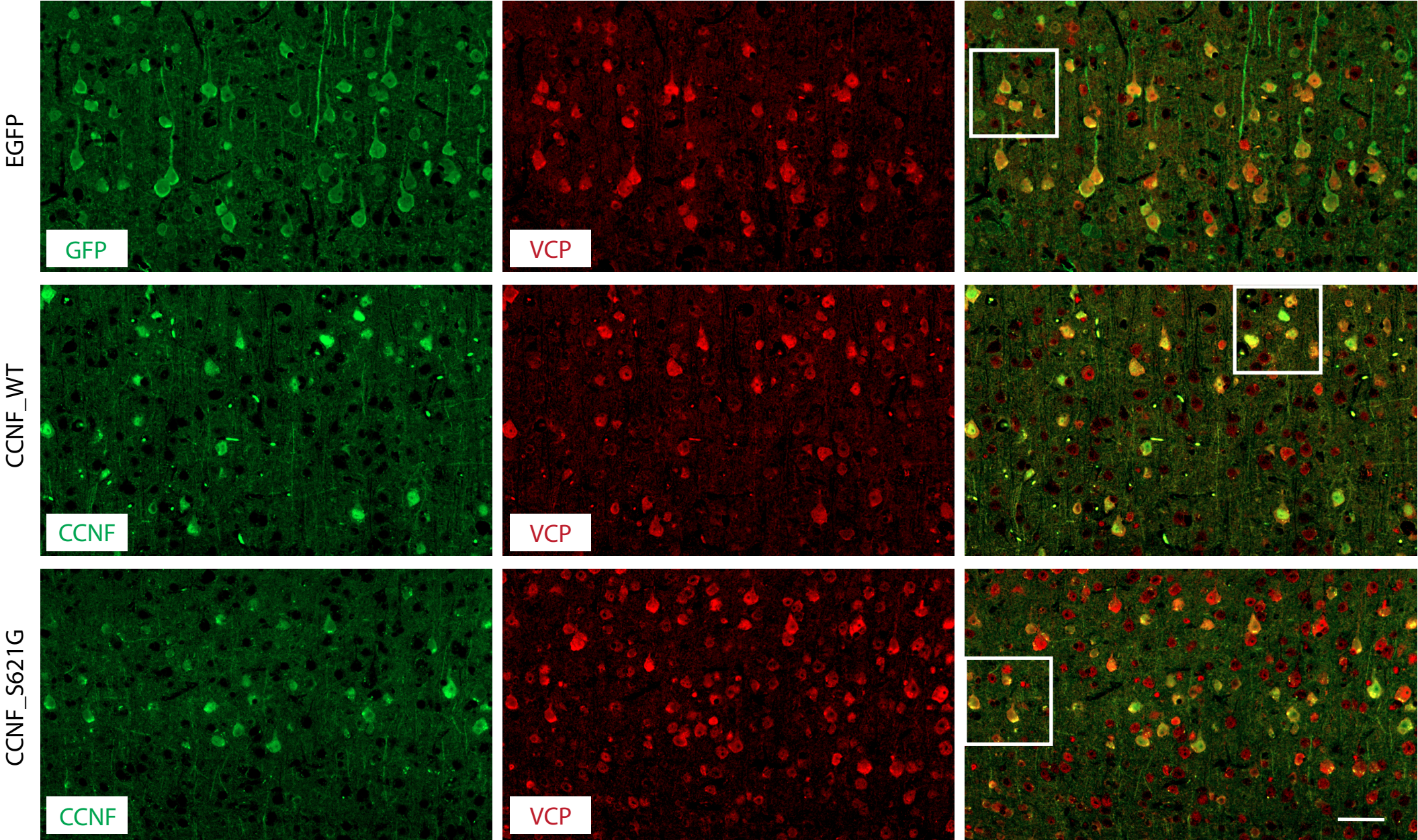

Supplement: Supplementary file 2 — Figure S2. Cytoplasmic inclusions in CCNF mice. Immunofluorescence of either GFP or CCNF double labelled with VCP antibodies in the cortices of 3‐month‐old control EGFP, CCNF_WT and CCNF_S621G. Boxed areas are depicted at higher magnification in Figure 5A. Scale bar 50 μm. [file NAN-49-0-s001.pdf]
